# Supplementary material for: The sequencing and interpretation of the genome obtained from a Serbian individual
Source: PLoS One. 2018 Dec 19;13(12):e0208901. doi: 10.1371/journal.pone.0208901 (PMC6300249; doi:10.1371/journal.pone.0208901)
Supplement: S1 File — (PDF) [file pone.0208901.s009.pdf]

Supporting Information: The sequencing and interpretation of the genome obtained from a Serbian individual

Wazim Mohammed Ismail[1*], Kymberleigh A. Pagel[1*], Vikas Pejaver[1], Simo V. Zhang[1], Sofia Casasa[2], Matthew Mort[3], David N. Cooper[3], Matthew W. Hahn[1,2], and Predrag Radivojac[4]

[1]Department of Computer Science, Indiana University, Bloomington, Indiana, U.S.A.
[2]Department of Biology, Indiana University, Bloomington, Indiana, U.S.A.
[3]Institute of Medical Genetics, Cardiff University, Cardiff, U.K.
[4]College of Computer and Information Science, Northeastern University, Boston, Massachusetts, U.S.A.

1 Read depth

The genome sequencing and mapping achieved an average read depth of 34.7, with 98.3% of GRCh37 reference bases having coverage of 10-fold or more and 89.4% having coverage of 20-fold or more. When only the non-zero positions were considered, the average read depth was 34.8, with 98.5% of GRCh37 reference bases having coverage of 10-fold or more and 89.7% having coverage of 20-fold or more. The number of zero-depth positions were 7,649,443 (0.3%). The depth of coverage distribution obtained using the two read mappers BWA and Bowtie2, before and after read processing - duplicate removal, base quality score recalibration and indel realignment, is shown in Supplementary Figure 1.

Figure 1: Histogram of depth of coverage obtained by two read mappers BWA and Bowtie2 before and after read processing - duplicate removal, base quality score recalibration and indel realignment.

2 Characterization of variant properties

Figure 2: Read depth across SNV and indel variants called by the four pipelines.

Figure 3: Size comparison of insertion and deletion variants across the four pipelines.

*Contributed equally to this work.

Table A: The total number of variants identified by four read mapper vs. variant caller platforms, collected from Figure 1 in the main paper. The results show significant variability of Platypus across two read mappers and also significant variability of Bowtie2 across two variant callers. We therefore selected a combination of BWA-MEM and GATK as our main platform for single nucleotide and short indel variants.

	**Bowtie2**	**BWA-MEM**
**Platypus**	2,850,611	3,381,575
**GATK**	3.248,823	3,280,675

Table B: Number of structural variants called by individual SV callers in SVE

**SV Caller**	**Deletion**	**Duplication**	**Insertion**	**Inversion**	**Translocation**	**Breakend**
BreakDancer	8489	903	7028	451	208	0
BreakSeq2	1993	0	140	0	0	0
cnMOPS	3931	257	0	0	0	0
CNVnator	10577	832	0	0	0	0
DELLY	12561	6969	943995	5469	62812	0
Hydra	5159	1337	0	76	0	176902
LUMPY	5759	550	0	61	0	2692
FusorSV	848	3	0	0	0	0

Figure 4: Number of heterozygous and homozygous SNV and indel variants called by the four pipelines.

Figure 5: Size comparison of indel variants in the Serbian genome.

3 Estimate of novel variation from the first individual of a newly sequenced population

Figure 6: The amount of novel variation observed in each 1000 Genomes Project participant compared to participants from other populations. The peak on the right side is comprised of individuals of African ancestry.

4 Length distribution of deletions called by FusorSV

Figure 7: Length distribution of deletions called by FusorSV.

Table C: Genes that overlap with deletions called by FusorSV.

A2ML1	*ABCB5*	*ABI3BP*	*ACACA*	*ACOT11*	*ACSL3*	*ADAM12*
ADCY8	*AHNAK2*	*AKAP6*	*AKT3*	*AL162389.1*	*AMDHD1*	*ANKRD30BL*
ANKRD55	*AP3S1*	*ARHGAP19*	*ARHGAP19-SLIT1*	*ASIP*	*ASMT*	*ATP10A*
ATP9B	*AVL9*	*BANK1*	*BCAR3*	*BCAS3*	*BCLAF1*	*BOP1*
BPGM	*C10orf11*	*C11orf49*	*C15orf57*	*C17orf85*	*C2CD2*	*C2orf73*
C8orf12	*CACNA1B*	*CACNB2*	*CASP8*	*CAST*	*CAV2*	*CCSER1*
CD2AP	*CDC40*	*CDH1*	*CDK19*	*CEP72*	*CHST9*	*CLASP2*
CLIC4	*CMSS1*	*CNOT10*	*CNTNAP2*	*CNTNAP4*	*COL23A1*	*COL5A2*
CSMD1	*CTD-2054N24.2*	*CTD-3105H18.18*	*CTNNA3*	*CYP2D6*	*CYP4F3*	*D2HGDH*
DAB1	*DACH2*	*DACT2*	*DCC*	*DENND1B*	*DIP2B*	*DKK2*
DLEU1	*DMPK*	*DNAJC27*	*DOCK5*	*DOCK8*	*DPF3*	*DPH7*
DPP6	*DYM*	*EDARADD*	*EGFR*	*EGLN3*	*EPHB1*	*EPSTI1*
ERC1	*ERICH1*	*ESR1*	*EXOC2*	*EYS*	*FAM149A*	*FAM154B*
FAM160A1	*FAM203B*	*FAM20C*	*FBXO36*	*FHIT*	*FILIP1L*	*FKBP5*
FMN2	*FRMD5*	*GABRG1*	*GALNT18*	*GALNTL6*	*GBP3*	*GDAP1*
GNG2	*GNGT1*	*GPR156*	*GRIK3*	*GRIN3A*	*HACE1*	*HCN2*
HHAT	*HIGD1A*	*HLA-A*	*HSF2BP*	*IFT52*	*IGHD1-20*	*IGHD2-21*
IGHD6-19	*IGHV4-61*	*IL2RA*	*IQCJ-SCHIP1*	*IQGAP3*	*KCNAB1*	*KCNIP1*
KCNJ12	*KIAA0391*	*KIAA1217*	*KLHL3*	*KLRC1*	*KLRC2*	*KM-PA-2*
KRT77	*KRTAP9-6*	*KRTAP9-7*	*LARS2*	*LCE3B*	*LCE3C*	*LEPREL1*
LGI1	*LHFP*	*LITAF*	*LRRN4*	*LTBP1*	*LUZP2*	*MACROD2*
MAGT1	*MAPK10*	*MATR3*	*METAP1D*	*METTL21A*	*METTL24*	*MGAM*
MGAT5	*MID1*	*MITF*	*MLIP*	*MROH1*	*MRPL42*	*MSI2*
MSR1	*MUC3A*	*MYCT1*	*MYO5B*	*MYO9A*	*NALCN*	*NBEAL1*
NCOA7	*NELL1*	*NKG2-E*	*NRG3*	*NRXN3*	*NT5M*	*OIP5*
OR4N4	*OR52N1*	*OR52N5*	*PADI4*	*PAPPA2*	*PARD3B*	*PARK2*
PC	*PCCB*	*PCDH15*	*PCDHB10*	*PCDHB16*	*PCDP1*	*PCM1*
PCSK5	*PDE4DIP*	*PDLIM3*	*PDSS1*	*PDXK*	*PEX5L*	*PHYHD1*
PIAS1	*PKHD1*	*PLEKHA7*	*POU6F2*	*PPAPDC1A*	*PPFIA2*	*PPM1B*
PPP1R1C	*PPP1R3A*	*PPP2R3B*	*PPP2R5A*	*PPP4R2*	*PRIM2*	*PRPSAP2*
PSD3	*PTPRD*	*PTPRK*	*PTPRN2*	*PTPRT*	*PWP2*	*RAD51B*
RALYL	*RAP1GAP2*	*RASGEF1B*	*RBPJ*	*RFPL4A*	*RIMS1*	*RNF144B*
RNF212	*RNF38*	*RP11-192H23.4*	*RP11-215A19.2*	*RP4-576H24.4*	*RPH3AL*	*RPIA*
RPTOR	*RTDR1*	*S1PR4*	*SAAL1*	*SBF2*	*SCAP*	*SCAPER*
SCML4	*SCNN1D*	*SCXA*	*SCXB*	*SERAC1*	*SERPINA1*	*SGCD*
SGK1	*SGSM3*	*SHISA9*	*SIRPB1*	*SLC12A8*	*SLC1A7*	*SLC25A18*
SLC35E4	*SLC4A10*	*SMYD3*	*SPAG16*	*SPOCK1*	*SPOCK3*	*SPRN*
STEAP2	*STON1*	*STON1-GTF2A1L*	*SUCLG2*	*SUMF1*	*SYT11*	*TBC1D16*
TBCE	*TCERG1L*	*TDG*	*THSD4*	*TJP2*	*TMBIM4*	*TMEM132D*
TMEM192	*TMEM41B*	*TOX2*	*TOX4*	*TPST1*	*TRAF3IP3*	*TRIM5*
TSPAN8	*TUBGCP2*	*UGT1A10*	*UGT1A4*	*UGT1A5*	*UGT1A6*	*UGT1A7*
UGT1A8	*UGT1A9*	*UGT2B17*	*UIMC1*	*UNC13B*	*UNKL*	*VMP1*
WDR34	*WDR47*	*WNK1*	*WWOX*	*WWTR1*	*XKR9*	*XRN2*
ZC3H11A	*ZDHHC19*	*ZMAT4*	*ZNF100*	*ZNF124*	*ZNF14*	*ZNF385D*
ZNF665	*ZPBP*					

Table D: Genes that overlap with duplications called by FusorSV.

IGHD1OR15-1A	*IGHD2OR15-2A*	*IGHD3OR15-3A*	*IGHD4OR15-4A*	*IGHD5OR15-5A*
IL9R	*PPP2R3B*	*SHOX*	*SPRY3*	*VAMP7*

5 Variants in genotype-phenotype annotation databases

5.1 HGMD annotations

There are five different classes of variant listed in HGMD. Disease-causing mutations (DM) are entered into HGMD where the authors of the corresponding report(s) have established that the reported mutation(s) are involved (or very likely to be involved) in conferring the associated clinical phenotype upon the individuals concerned. The DM classification may, however, also appear with a question mark (DM?), denoting a probable/possible pathological mutation, reported as likely to be disease causing in the corresponding report, but where (i) the author has indicated that there may be some degree of doubt or uncertainty; (ii) the HGMD curators believe greater interpretational caution is warranted, or (iii) subsequent evidence has appeared in the literature which has called the initial putatively deleterious nature of the variant into question (e.g. a negative functional, case–control or population-scale sequencing study). In addition, three categories of polymorphism are included in the database. Disease-associated polymorphisms (DP) are entered into HGMD where there is evidence for a significant association with a disease/clinical phenotype along with additional evidence that the polymorphism is itself likely to be of functional relevance (e.g., as a consequence of genic location, evolutionary conservation, transcription factor binding potential, etc.), although there may be no direct evidence (e.g., from an expression study) for a functional effect. The functional polymorphisms (FP) class includes those sequence changes for which a direct functional effect has been demonstrated (e.g., by means of an *in vitro* reporter gene assay or alternatively by protein structure, function or expression studies), but with no disease association reported as yet. Disease-associated polymorphisms with supporting functional evidence (DFP) must meet both of the above criteria in that the polymorphism should not only have been reported to be significantly associated with disease, but should also display direct evidence of being of functional relevance.

5.2 ClinVar annotations

Several variants within the Serbian genome are characterized with pathogenic Clinical Significance in the ClinVar database. Nine variants are observed in the homozygous state in the Serbian genome which correspond to "Pathogenic" Clinical significance in ClinVar. Of these, seven have been submitted without assertion criteria and represent less confident annotation. The remaining two entries are provided by a single submitter and are similarly not of sufficient confidence to be included explicitly in the manuscript.

There are 25 heterozygous variants annotated as "Pathogenic" in the Serbian genome. Of these, many are low confidence: 11 are submitted without assertion criteria, 4 have criteria provided by a single submitter, and 3 have conflicting interpretations. The remaining 7 variants have criteria provided by multiple submitters without conflicting interpretations. The heterozygous variants are associated with multiple phenotypes, including trimethylaminuria, irido-corneo-trabecular dysgenesis, glaucoma, anterior segment dysgenesis, CYP1B1-related disorders, progressive familial heart block, skin and nail abnormalities, epidermolysis bullosa, butyrylcholine esterase deficiency, postanesthetic apnea, hereditary cancer-predisposing syndromes, and Niemann-Pick disease. Of these phenotypes, none are known to have occurred

Table E: Frameshifting insertion/deletion variants scored with MutPred-LOF

Gene	Variant	Score	Affected Molecular Mechanisms
NM_030665	Q280Hfs*84	0.57394	Methylation(p=0.003); Amidation(p=0.004); Ubiquitylation(p=0.004); MoRF(p=0.005); O-linked glycosylation(p=0.007)
NM_030665	Q280Afs*108	0.57334	Methylation(p=0.003); Amidation(p=0.004); Ubiquitylation(p=0.004); MoRF(p=0.005); O-linked glycosylation(p=0.007)
NM_024675	R170Ifs*14	0.54289	MoRF(p=0.03); Pyrrolidone carboxylic acid(p=0.033); Loop(p=0.034); Amidation(p=0.036); O-linked glycosylation(p=0.038)
NM_006580	A56Lfs*16	0.52228	Palmitoylation(p=0.02)
NM_001037333	Q95Pfs*15	0.511	Helix(p=0.015); PPI hotspot(p=0.038)
NM_001035235	V110Dfs*2	0.50882	NA
NM_001009931	M1Cfs*28	0.50536	Signal cleavage(p=0); RNA binding(p=0); Sulfation(p=0); B factor(p=0.0001); Copper binding(p=0.0002)

within the immediate family history.

Table H: Top scoring noncoding variants from CADD

Chr	Pos	Ref/Alt	Consequence	Score
17	59667953	G/A	noncoding_change	37
16	88780634	AGGTGTG/A	downstream	35
2	85570766	CAGAT/C	upstream	35
17	40270029	ACTGAAGCTGAGGAGAGAGAGAGACGT CAGGGATGGGGGG/A	downstream	27
8	145617534	TGGGGGTGCAAGGTGA/T	downstream	25.3
17	59668021	G/C	noncoding_change	24.4
22	37465385	CTGGGG/C	regulatory	24.4
14	77648237	TGTG/T	regulatory	24.3
2	61389737	T/G	noncoding_change	23.8
19	44610843	C/T	noncoding_change	23.8
6	160560897	CTGGTAAGT/C	regulatory	23.5
2	20448521	C/T	upstream	23.5
3	10368577	C/A	upstream	23.3
12	104341644	AACTG/A	downstream	23.2
3	6513628	CACAG/C	intergenic	23.2
3	96336236	G/T	upstream	23.2
9	113128561	TGAGGGTAG/T	downstream	23.1
15	37172064	C/A	downstream	23.1
4	8785222	C/G	intergenic	23.1
7	11874349	TA/T	upstream	23.1
17	36782034	TAAA/T	upstream	23.1
21	35795163	T/A	upstream	23.1
4	24516813	TTTATC/T	downstream	23
7	131185995	C/A	downstream	23
10	103002063	G/A	downstream	23
11	61165731	C/CA	downstream	23
15	38982825	C/CT	downstream	23

15	100514418	C/G	downstream	23
17	5286861	GTAGTGTTTGGAATTTTCTGTTCATA/G	downstream	23
1	91621787	TAACA/T	intergenic	23
4	156503230	ATATT/A	intergenic	23
5	130484813	T/A	intergenic	23
6	14806096	GTGACAT/G	intergenic	23
6	85335098	T/A	intergenic	23
9	96708343	A/G	intergenic	23
11	36910028	TAA/T	intergenic	23
14	25819285	CAT/C	intergenic	23
21	16468682	TACAA/T	intergenic	23
3	64430551	G/C	upstream	23
6	32083175	C/T	upstream	23
10	62493816	G/A	upstream	23
11	93584075	T/A	upstream	23
12	114843606	C/T	upstream	23
16	87638576	G/A	upstream	23
19	47991316	TTAAA/T	upstream	23
5	123647724	GACA/G	downstream	22.9
5	139749184	G/A	downstream	22.9
7	27197601	C/G	downstream	22.9
8	80678427	T/C	downstream	22.9
11	61513671	CAGAG/C	downstream	22.9
11	125830697	T/C	downstream	22.9
12	48396920	G/T	downstream	22.9
15	100514417	C/T	downstream	22.9
16	31000726	TAGAAATGGGACTCTGAGGGCTAAC/T	downstream	22.9
1	41314741	CA/C	intergenic	22.9
1	92081030	T/A	intergenic	22.9
1	96499830	T/A	intergenic	22.9
2	216776870	T/A	intergenic	22.9
3	6513635	CGACA/C	intergenic	22.9
3	44148568	ATGCT/A	intergenic	22.9
4	31809946	C/CACAC	intergenic	22.9
5	31024929	TA/T	intergenic	22.9
6	40146909	ATAAAT/A	intergenic	22.9
7	9863374	TA/T	intergenic	22.9
7	27252670	A/G	intergenic	22.9
10	86871665	TA/T	intergenic	22.9
11	42520473	T/A	intergenic	22.9
15	95252667	T/A	intergenic	22.9
1	40155486	A/G	upstream	22.9
1	48965535	TAAAC/T	upstream	22.9
2	219722664	T/C	upstream	22.9
5	117901084	T/A	upstream	22.9
7	20373528	TTA/T	upstream	22.9
7	44618599	T/A	upstream	22.9
9	107694522	C/T	upstream	22.9
11	63869107	T/A	upstream	22.9
1	3801973	T/C	downstream	22.8
4	61659482	C/CCTT	downstream	22.8

Figure 8: Gene Ontology (GO) terms enriched in the set of 81 genes that harbored the 95 missense variants predicted to be pathogenic. Within the three domains, the top ten terms with significant enrichment are shown in red (P<0.05), terms with non-significant enrichment are in dark red, and the ancestor terms are shown in black.

5	123799069	C/G	downstream	22.8
5	156186841	G/A	downstream	22.8
7	115147752	C/A	downstream	22.8
8	28911173	AGACT/A	downstream	22.8
9	127642032	CAT/C	downstream	22.8
12	6054748	T/C	downstream	22.8
12	53806714	CTG/C	downstream	22.8
14	35903276	GGACATTTA/G	downstream	22.8
15	92716979	T/G	downstream	22.8
2	4155192	T/A	intergenic	22.8
2	143562694	T/G	intergenic	22.8
2	222173880	T/A	intergenic	22.8
4	83012112	T/A	intergenic	22.8
4	83119414	T/A	intergenic	22.8
5	107795557	T/A	intergenic	22.8
5	116888166	T/A	intergenic	22.8
5	166025268	C/T	intergenic	22.8
7	25314445	T/A	intergenic	22.8
10	59358896	T/A	intergenic	22.8
9	75829027	T/A	intergenic	22.8
9	116544406	T/G	intergenic	22.8
11	58232952	T/A	intergenic	22.8

Table F: Non-frameshifting insertion/deletion variants scored with MutPred-Indel

**Gene**	**Variant**	**Score**	**Affected Molecular Mechanisms**
NM_000183	T2_I3insT	0.78938	NA
NM_147196	K131Dfs*26	0.69025	NA
NM_001085451	S80_H81insSDRLPRRHSHEDQEFRCRS	0.70536	NA
NM_000492	L454Afs*1027	0.81268	NA
NM_007343	E89Nfs*216	0.70583	NA
NM_001304441	V191Afs*254	0.68519	Mn binding (p=0.005); PPI hotspot (p=0.006); Transmembrane region (p=0.008); PPI residue (p=0.011); Strand (p=0.016)
NM_000423	S101_F102insG	0.6849	NA
NM_003771	S458Rfs*10	0.71099	NA
NM_002144	A2_P28insHSA	0.77485	NA
NM_138690	A1028Gfs*8	0.69605	NA
NM_002152	D261Vfs*439	0.68652	NA
NM_002152	E204_A205insE	0.68487	NA

Table G: Splice variants scored with MutPred Splice

Gene	Variant	Genomic Coordinates	Score	Confident Hypotheses
NP_001091994.1	I229T	chr19,17648350,+	0.68	
NP_775815.2	I229T	chr19,17648350,+	0.68	
NP_115702.1	Q65P	chr4,944210,+	0.78	
NP_001727.1	D2N	chr19,47823038,+	0.8	Loss of natural 3' SS
NP_702918.1	T218T	chr9,131235321,+	0.81	
NP_702915.1	T218T	chr9,131235321,+	0.81	
NP_002531.3	T213T	chr9,131235321,+	0.81	
NP_702914.1	T237T	chr9,131235321,+	0.81	
NP_702917.1	T262T	chr9,131235321,+	0.81	
NP_702913.1	T301T	chr9,131235321,+	0.81	
NP_001229281.1	T232T	chr9,131235321,+	0.81	
NP_001229283.1	T156T	chr9,131235321,+	0.81	
NP_702911.1	T237T	chr9,131235321,+	0.81	
NP_001229282.1	T237T	chr9,131235321,+	0.81	
NP_702910.1	T281T	chr9,131235321,+	0.81	
NP_000322.2	T77S	chr11,18290880,+	0.83	
NP_954630.1	T77S	chr11,18290880,+	0.83	
NP_001171477.1	T77S	chr11,18290880,+	0.83	
NP_065946.2	A357A	chr19,35506729,+	0.86	Cryptic 3' SS
NP_001129671.1	A350A	chr19,35506729,+	0.87	Cryptic 3' SS
NP_001018101.1	Q175Q	chr15,57918090,+	0.87	
NP_001018100.1	Q175Q	chr15,57918090,+	0.87	
NP_689664.3	Q175Q	chr15,57918090,+	0.87	
NP_001018110.1	Q175Q	chr15,57918090,+	0.87	
NP_055146.1	S481S	chr4,139100372,-	0.88	
NP_001185763.2	A1757T	chr1,144868170,-	0.91	Cryptic 5' SS
NP_055459.4	A1757T	chr1,144868170,-	0.91	Cryptic 5' SS
NP_001185761.1	A1651T	chr1,144868170,-	0.91	Cryptic 5' SS

Table I: Top 5 diseases enriched in the set of 83 genes containing predicted pathogenic missense variants

Disease term	Number of genes	Entrez gene IDs	Benjamini-Hochberg adjusted P-value
Renal diabetes	5	55748, 1636, 2646, 9844, 6580	0.0005
Cardiovascular Diseases	8	4153, 350, 10060, 3816, 1636, 4624, 2646 2153	0.0007
Ischemia	5	4153, 10060, 9620, 1636, 2153	0.0007
Respiratory Tract Diseases	7	4153, 27294, 5270, 1285, 1636, 27159 2153	0.0007
Lung Diseases	7	4153, 27294, 5270, 1285, 1636	0.0007
